# Automatic lung nodule detection using multi-scale dot nodule-enhancement filter and weighted support vector machines in chest computed tomography

**DOI:** 10.1371/journal.pone.0210551

**Published:** 2019-01-10

**Authors:** Yu Gu, Xiaoqi Lu, Baohua Zhang, Ying Zhao, Dahua Yu, Lixin Gao, Guimei Cui, Liang Wu, Tao Zhou

**Affiliations:** 1 School of Computer Engineering and Science, Shanghai University, Shanghai, China; 2 Inner Mongolia Key Laboratory of Pattern Recognition and Intelligent Image Processing, School of Information Engineering, Inner Mongolia University of Science and Technology, Baotou, Inner Mongolia, China; 3 School of Foreign Languages, Inner Mongolia University of Science and Technology, Baotou, Inner Mongolia, China; Beijing University of Technology, CHINA

## Abstract

A novel CAD scheme for automated lung nodule detection is proposed to assist radiologists with the detection of lung cancer on CT scans. The proposed scheme is composed of four major steps: (1) lung volume segmentation, (2) nodule candidate extraction and grouping, (3) false positives reduction for the non-vessel tree group, and (4) classification for the vessel tree group. Lung segmentation is performed first. Then, 3D labeling technology is used to divide nodule candidates into two groups. For the non-vessel tree group, nodule candidates are classified as true nodules at the false positive reduction stage if the candidates survive the rule-based classifier and are not screened out by the dot filter. For the vessel tree group, nodule candidates are extracted using dot filter. Next, RSFS feature selection is used to select the most discriminating features for classification. Finally, WSVM with an undersampling approach is adopted to discriminate true nodules from vessel bifurcations in vessel tree group. The proposed method was evaluated on 154 thin-slice scans with 204 nodules in the LIDC database. The performance of the proposed CAD scheme yielded a high sensitivity (87.81%) while maintaining a low false rate (1.057 FPs/scan). The experimental results indicate the performance of our method may be better than the existing methods.

## Introduction

Lung cancer is a serious public health problem in the world. Lung cancer prevalence estimates for 5 years was over 884,000 cases in 2011, which is the third most prevalent cancer after breast cancer and colorectal cancer in China[1]. Five-year survival of lung cancer is 16.1% in China[2], Seventeen per cent in the United States[3] and 13% in Europe[4]. If the lung nodule is detected in the earlier stages of lung cancer, the overall 5-year survival rate can increase to 55%[5, 6]. Therefore, screening programs for early detection and diagnosis of lung cancer have been attempted in many countries, which is designed to allow patients to be treated early enough to reduce lung cancer mortality[7]. According to the National Lung Screening Trial, low dose computed tomography (LDCT) screening can reduce lung cancer mortality by 20% compared with chest x-ray screening[8, 9]. In a screening program with LDCT, radiologists must read many medical images and are likely to overlook some subtle nodules which could be lung cancers. Therefore, computer-aided detection (CAD) schemes, which can provide the locations of nodule candidates, serve as a “second opinion” to aid the radiologists in making faster and more accurate diagnoses.

Juxta-vascular nodules are challenging nodules, which are often missed by CAD systems[10]. Thus, the proposed scheme aimed to detect lung nodules, especially juxta-vascular nodules. Furthermore, it was found that it was much more difficult for CAD systems to detect juxta-vascular nodules attached to tiny vessels than those attached to large vessels. Meanwhile, isolated nodules and juxta-pleural nodules usually did not appear in the vessel tree group. Therefore, two nodule candidate groups—the non-vessel tree group and the vessel tree group—were formed to detect lung nodules.

This paper contains two main innovations. The first innovation is that juxta-vascular nodules attached to tiny vessels are detected in the non-vessel tree group for the first time; also, instead of a uniform threshold, different thresholds are used to extract juxta-vascular nodules attached to the vessel tree or tiny vessels when nodule-enhanced image obtained from dot filter is binarized. Not only surface gradient features, but also shell-based gradient features are extracted. As surface gradient features are susceptible to the accuracy of nodule segmentation, shell-based gradient features combined with surface gradient features can improve the classification accuracy when discriminating juxta-vascular nodules from vessel bifurcations.

## Related work

The definition for nodule by thoracic CT based on the Fleischner’s Society is “a round opacity that is at least moderately well marginated and no greater than 3 cm in maximum diameter”[11]. Many researchers developed schemes to detect lung nodules. Wu et al[12] developed a technique based on the thresholding method and region growing algorithm to obtain nodule candidates. Next, false positives were removed by using invariant moments. However, some vessels cut by the segmentation procedure of ROI could be misclassified as nodules since they have similar shapes. S.Sivakumar et al.[13] adopted weighted fuzzy-possibilistic C-Means combined with a SVM classifier to detect nodules. As the clustering methods are threshold-based methods, these types of methods may not detect ground-glass lung nodules. Ayman et al.[14] proposed a deformable 3D and 2D templates-matching method to detect nodules. It may be hard to detect some small nodules by this method because they are often confused with bronchioles and small blood vessels. Li et al.[7, 15] constructed selective enhancement filters to detect nodules. However, the scheme they proposed could not distinguish the juxta-vascular nodules from the vessel bifurcations because with the intensity of the vessel bifurcations changed by Gaussian smoothing, it was transformed into a blob-like structure; this led to incorrect enhancement at these bifurcation regions. Chen et al.[16] proposed a method of local intensity structure analysis combined with front surface propagation for nodule detections. Their method had satisfactory performance, and it would be beneficial to verify the algorithm on large data sets. Riccardi et al.[17] proposed a 3D fast radial filter to detect nodules. Then, false positives were removed by using a heuristic FPR method and a supervised FPR method. They demonstrated outstanding performance for nodule detection, but the overall performance of their scheme could be further improved by removing some false positives close to the pleura with specific methods. Li et al.[18] proposed a two-stage classification approach using rule-based and C-SVM classifiers for detecting both solid nodules and ground-glass opacity (GGO) nodules. Their method can be further improved if 3D features can be further extracted and an adaptive smoothing method can be further investigated to deal with image noise. Tan et al.[19] applied the Feature-Deselective Neuro-Evolving Augmenting of Topologies (FD-NEAT) classifier to discriminate lung nodules from false positives. This bypassed the need to pre-define the topology of the neural networks, which also incorporated the feature selection into the classification step. GGO nodules were not considered in their research. Setio et al.[20] adopted multi-view convolutional networks to detect lung nodules. The training data were multi-planner views of CT scans. The overall performance of their scheme may be further increased if the candidate detection algorithm is improved. Javaid et al.[21] divided nodule candidates into six groups based on their thickness and extracted different features from nodules in each group to eliminate false positives (FPs), then an SVM classifier was used for classification. The performance of GGO nodule detection may be further improved. Filho et al.[22] adopted quality threshold clustering, genetic algorithms and diversity indices to detect solitary lung nodules. The performance of their method may be further improved when dealing with juxta-vascular nodules. Gong et al.[23] combined 3D tensor filtering with local image feature analysis to detect lung nodules. As their method is based on the hypothesis that the nodules have ball-like or dot-like structures, some irregularly shaped nodules may be omitted by their scheme. Torres et al.[24] extracted a set of 13 features for nodule candidate analysis, including intensity, spatial and shape features. Then, they proposed a feed-forward artificial neural network (FFNN) to classify the candidates. The performance of GGO and subtle nodule detection may be further improved.

## Methods

### Overview

**Fig 1:**
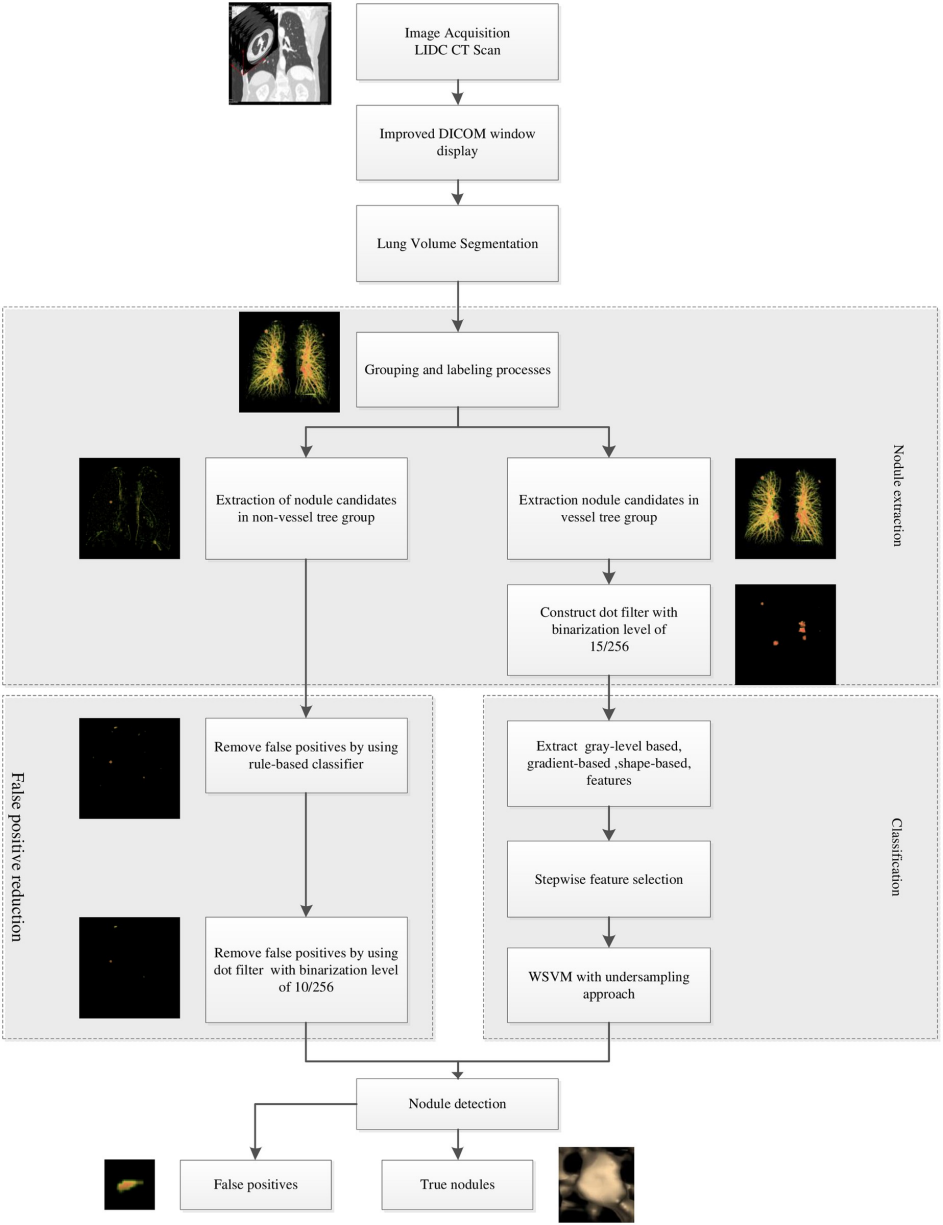
The overall block diagram of the proposed CAD scheme.

In this section, the proposed CAD scheme is described. The proposed CAD scheme contains four major steps: lung segmentation, nodule candidate extraction, reduction of false positives and classification. The overall diagram of the proposed CAD scheme is displayed in Fig 1.

### Improved DICOM window display technology

Before lung segmentation was performed, the chest CT scans were displayed with lung (width, 1600 HU; level,-600 HU) windows instead of mediastinal windows (width, 400 HU; level, 20HU) because the area of GGO—corresponding to the area of the bronchioloalveolar carcinoma (BAC) component of the lung adenocarcinomas—disappears when the images are viewed via mediastinal window in pathologic correlative studies[23]. Then, the pixel value of CT scans was converted to 0–1 based on the lung window. Decimals—rather than 256 level gray scales—were used to represent gray intensity to avoid rounding errors.

### Lung segmentation

The lung segmentation was performed to identify the boundaries of the lungs as a prerequisite step for lung nodule detection[25, 26]. The precise segmentation of lung regions is a very crucial step because it ensures that the lung nodules—especially juxta-pleural nodules—are not missed due to inaccurate segmentation; also, it restricts subsequent processes to the lung regions in order to exclude FPs outside the lung region as much as possible. Many state-of-the-art lung segmentation methods have been proposed in recent years for the early diagnosis of lung cancer[27–31]. Filho et al.[28] proposed a novel and powerful 3D adaptive crisp active contour (3D ACACM) method for lung segmentation. The proposed 3D ACACM method obtained higher performance levels than watershed, region growing, mathematical morphology and conventional active contour techniques. Furthermore—on the basis of ACACM lung volume segmentation—they adopted an optimum-path forest classifier to identify lung fibrosis structures and Chronic Obstructive Pulmonary Disease (COPD)[32]. Zhang et al.[29] proposed a novel region- and edge-based geometric active contour (REGAC) model for lung segmentation, which improved segmentation accuracy when dealing with lung regions with weak boundaries. Soliman et al.[30] adopted an adaptive appearance-guided shape modelling method to segment pathological lungs. Hosseini et al.[31] applied a novel incremental constrained non-negative matrix factorization (ICNMF)-based lung segmentation method, which extracted voxel-wise features by using a few parameters. All these methods mentioned above have achieved satisfactory results for lung volume segmentation.

**Fig 2:**
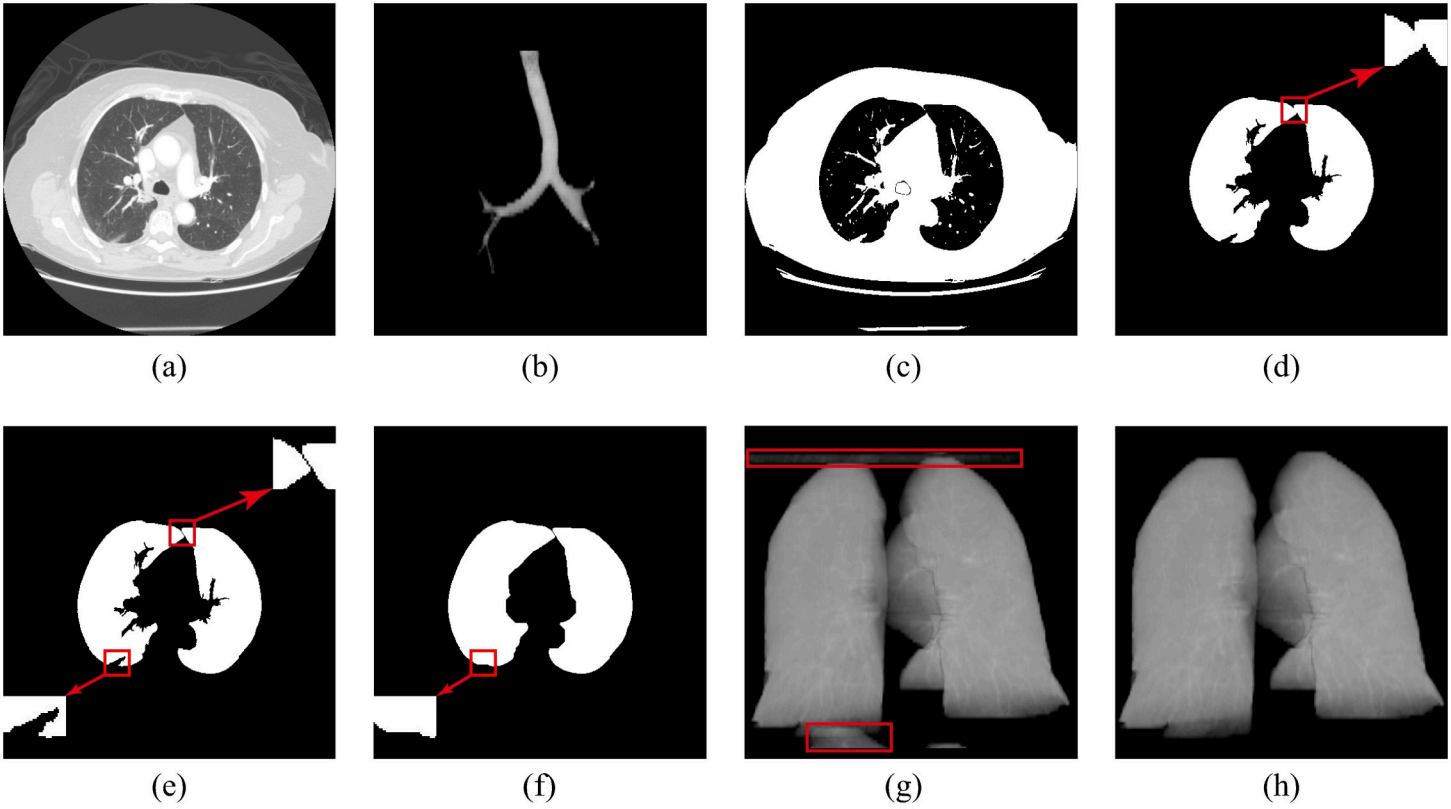
Lung volume segmentation steps. **a)** Original image, **b)** 3D image of the main trachea and bronchus tree, **c)** binary image in which the main trachea and bronchus tree were filled, **d)** initial segmented lung regions and local enlarged view of the fused position, **e)** fused lung region separated into two distinct regions and local enlarged view of indentation location, **f)** boundary repairing, **g)** 3D image of lung volume with artifacts and other tissue and **h)** 3D image of lung after removing other structures.

In this research, we proposed a concise and accurate lung volume segmentation method for lung nodule detection. The proposed lung volume segmentation method consists of four substeps, shown in Fig 2: (1) the initial lung region was extracted by using Otsu’s method, (2) the main trachea and bronchus tree was removed by adopting 3D region growing technology, (3) the fused lung region was separated into two distinct regions after automated location of the anterior junction line—if it exists—by utilizing the gray integral projection method and (4) the indentations along the lung contour lines were filled by utilizing rolling ball technology[33]. When the indentations were filled, a ball was tangentially placed and then rolled along the lung contour lines. Indentations were filled when the ball contacted the lung contour lines at more than one point. The radius of the ball was set experimentally to 15 mm, which was suitable to most of the conditions, as the nodules are no greater than 3 cm in maximum diameter.

The processing procedure for lung volume segmentation is shown in Fig 2, in which picture (a) is the original chest CT image, picture (b) illustrates the 3D view of the extracted main trachea and bronchus tree, picture (c) represents the binary image after the main trachea and the bronchus tree are filled, picture (d) shows the initial segmented lung regions and the local enlarged view of the fused position, picture (e) demonstrates the fused lung region which is separated into two distinct regions and the local enlarged view of the indentation location, picture (f) is the result of boundary repair, picture (g) is the 3D image of the lung volume with artefacts and other tissue and picture (h) shows the final result of the segmented lung volume after removing other structures.

### Extraction of nodule candidates

**Fig 3:**
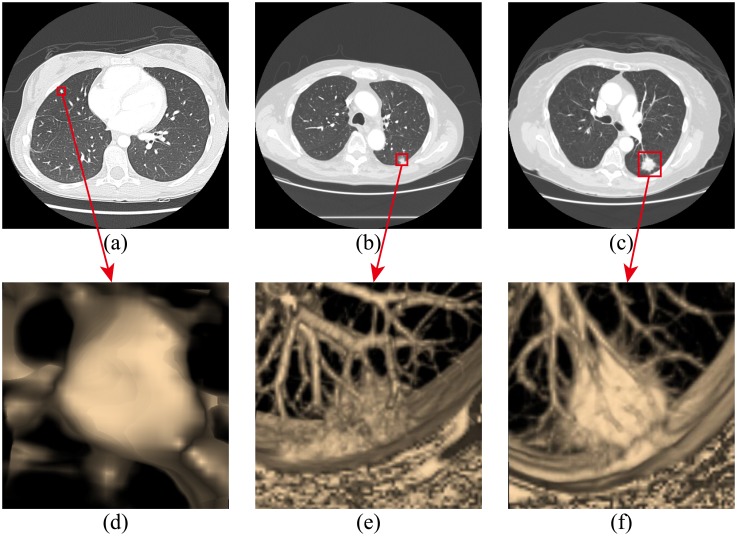
Three types of lung nodules. **a)** isolated nodule, **b)** 3D image of isolated nodule, **c)** juxta-pleural nodule, **d)** 3D image of juxta-pleural nodule, **e)** juxta-vascular nodule and **f)** 3D image of juxta-vascular nodule.

Three types of lung nodules were detected, including isolated nodules, juxta-pleural nodules and juxta-vascular nodules. Juxta-vascular nodules may also be attached to pleural tissues, as shown in Fig 3. In Fig 3, the pictures (a), (b), and (c) respectively represent the isolated nodule, juxta-pleural nodule and juxta-vascular nodule; the pictures (d), (e), and (f) show the 3D view of the pictures (a), (b), and (c). Extraction of nodule candidates in the proposed scheme requires three substeps: grouping and labeling processes; extraction of nodule candidates attached to the vessel tree in the vessel tree group; and extraction of isolated nodule candidates, juxta-pleural nodule candidates, and juxta-vascular nodule candidates attached to tiny vessels in the non-vessel tree group.

#### Grouping and labeling processes

**Fig 4:**
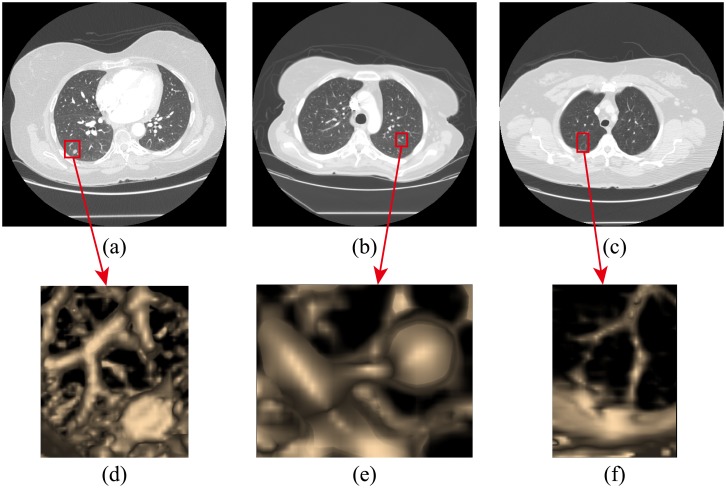
Examples of juxta-vascular nodules attached to tiny vessels. **a)**, **c)** and **e)** are images that contain juxta-vascular nodules in the box. **b)**, **d)** and **f)** are 3D views of juxta-vascular nodules related to juxta-vascular nodules in images **a)**, **b)** and **c)**, respectively.

After the lung region was obtained, the vessels and nodule candidates were extracted by the improved Otsu’s method. It was improved to calculate the multilevel thresholds of chest CT scans. Then, the 3D labeling technology with 26-neighborhood was performed to generate a 3D data set. In each lung, the largest connected structure corresponds to the pulmonary vessel tree. The juxta-vascular nodules attached to the pulmonary vessel tree were also included in this vessel tree group. The remaining structures were labeled as the non-vessel tree group. However, with the utilization of Otsu’s method, tiny blood vessels probably attached with juxta-vascular nodules—which are tiny branches at the end of the vascular tree—would be displayed as not connecting with the pulmonary vessel tree; the gray intensity of some parts of the tiny blood vessel is near to that of pulmonary parenchyma, as shown in Fig 4. In Fig 4, the pictures (a), (b) and (c) are three examples of these components mentioned above, while the pictures (d), (e) and (f) are the 3D views of the pictures (a), (b) and (c), respectively. Thus, the remaining structures were labeled as the non-vessel tree group—which contained isolated nodules—juxta-pleural nodules, tiny blood vessels, juxta-vascular nodules attached to tiny blood vessels, other kinds of lesions, and noise. The false positives in the non-vessel tree group were removed at the false positives step.

#### Extraction of juxta-vascular nodule candidates attached to the vessel tree

Extraction of juxta-vascular nodules is difficult because juxta-vascular nodules are attached to the pulmonary vessels and the gray intensity of them is close. Thus, the dot-shape selective enhancement filter proposed by Li et al.[7, 15] was applied to cut away the juxta-vascular nodules from the blood vessels attached.

The dot-shape selective enhancement filter was used not only to distinguish the spherical structure from the tubular structure or planar structure but also to cut away the juxta-vascular nodules from the blood vessels attached, shown in Eq 1.

$$d(x,y,z)=\text{exp}(-\frac{x^{2}+y^{2}+z^{2}}{2\sigma^{2}})$$ (1)

In Eq 1, *d*(*x*, *y*, *z*) is a ‘‘fuzzy” dot in the form of a 3D Gaussian function[7, 15].

The dot-shape selective enhancement filter was constructed by use of Eq 2.

$$Z_{dot}(\lambda_{1},\lambda_{2},\lambda_{3})={\left|\lambda_{3}\right|}^{2}/\left|\lambda_{1}\right|,if\lambda_{1}<0,\lambda_{1}<0,\lambda_{1}<0;\begin{array}{ccc} & 0 & otherwise \end{array}$$ (2)

In Eq 2, *λ*_1_, *λ*_2_ and *λ*_3_ are the three eigenvalues of the Hessian matrix, which satisfy |*λ*_1_|≥|*λ*_2_|≥|*λ*_3_|.

The dot-shape selective enhancement filter was used to enhance the spherical shapes and to suppress other shapes in the vessel tree group[34]. Thus, the nodule-enhanced image of the vessel tree group was obtained. Then, the nodule-enhanced image of the pulmonary vessels tree group was given a threshold with the parameter *T*_*nodulemask_vesseltreegroup*_. The proposed scheme detects subtle nodules, though the vessel bifurcations are also enhanced. The vessel bifurcations are addressed in the classification method introduced in section “Classification of lung nodules in the vessel tree group using WSVM with imbalance data”.

Then, a 3D labelling technique was used to identify the isolated objects in nodule-enhanced images with 26-neighborhood. Different from Li et al., we did not eliminate small objects because small nodules would be removed along with false positives. As the nodule candidates obtained from the nodule-enhanced image appear slightly smaller, a 3D constrained region-growing technique was performed to constrain growth up to 5 mm. So far, the juxta-vascular nodule candidates in the vessel tree group were extracted.

However, the gray intensity of vessel bifurcations change when the image is convolved with Gaussian filter so that the vessel bifurcations are similar to blob structures, which may be enhanced by dot-shape enhancement filter incorrectly[16]. The method for eliminating vessel bifurcations will be introduced in the subsequent sections.

### False positives reduction for nodule candidates in non-vessel tree group

#### Remove obvious false positives by using a rule-based classifier

Firstly, rule-based classifiers are designed to remove obvious false positives. The volume of the candidates was constrained first. As nodules range in size from 3–30 mm, a nodule candidate with a volume larger than 14.14 cm^3^ (30 mm in maximum diameter) should be considered a mass or non-nodule. Similarly, a nodule candidate with a volume smaller than 14.14 mm^3^ should be considered a non-nodule or background noise.

Considering that nodules are typically spherical and compact, three features were extracted from nodule candidates to eliminate obvious false positives.

The elongation factor of each candidate was computed as the distance ratio of the major axis to the minor axis of a minimum bounding ellipse in transverse section, shown as Eq 3.

$${\text{R}}_{\text{elongationFactor}}\;=\text{majorAxisLength}/\text{minorAxisLength}$$ (3)

In Eq 3, majorAxisLength and minorAxislength denote the length of the major and the minor axis, respectively.

The compactness of each candidate was computed as the ratio of its area to the area of the smallest bounding box in each transverse section, shown as Eq 4.

$${\text{R}}_{\text{compactness}}\;=\text{area}/\text{areaofboundingbox}$$ (4)

In Eq 4, area and areaofboundingbox denote the area of each candidate and its smallest bounding box, respectively.

The feature of sphericity was calculated as the proportion of structure volume included within a sphere of equivalent volume centered at the structure’s center of mass[33], shown as Eq 5.

$${\text{R}}_{\text{sphericity}}\;=\left(S\cap C\right)/S$$ (5)

In Eq 5, S is the set of voxels encompassed by the structure in all transverse sections and C is the set of voxels encompassed by the volume-equivalent sphere. A candidate was also eliminated if sphericity of the candidate was less than 0.3.

A candidate was not considered to be a nodule and then was eliminated if R_elongationFactor_ > 6, or R_compactness_ < 0.3, or R_sphericity_ < 0.3.

The cut-off thresholds of these three features were very lax since we found that juxta-vascular nodules attached to tiny vessels were in the non-vessel tree group as mentioned above. Those thresholds were set experimentally to ensure juxta-vascular nodules attached to tiny vessels would not be eliminated.

#### Remove false positives by using dot filter

To further remove the false positives and to separate juxta-vascular nodules from tiny vessels, the dot filter was used again to extract the spherical shapes and to suppress other shapes in the non-vessel tree group. Then, the nodule-enhanced image from the non-vessel tree group was compared to the threshold using the parameter *T*_*nodulemask_non-veseltreegroup*_. The bifurcations of the tiny vessels—which were also enhanced by dot filter—would not be selected because the response by the dot filter was weaker than that of the vessel tree group when the parameter *T*_*nodulemask_non-veseltreegroup*_ was set appropriately. The proposed scheme detects subtle nodules, although it also enhances vessel bifurcations. Meanwhile, the juxta-vascular nodule is not missed. Then, objects smaller than 4.19 mm^3^ (2 mm in diameter) were not considered to be nodules and were removed. Finally, the constrained region growing technique was adopted to get a maximum of 5 mm of growth. The nodule candidates that survived the rule-based classifier and not screened out by the dot filter were classified as nodules.

#### Features extraction of juxta-vascular nodule candidates in the vessel tree group

Features were calculated based on the gray-level, gradient and shape to form the feature pool.

#### Gray-level based features

As nodules often have higher CT attenuation than false positives caused by vessel bifurcation, four three-dimensional gray-level based features were extracted: 1) maximum value of gray level (*gray_level_max*), 2) minimum value of gray level (*gray_level_min*), 3) mean value of gray level (*gray_level_mean*), and 4) standard deviation of gray level (*gray_level_std*).

#### Gradient-based features

The gray-level distribution of nodules is approximately symmetric radially, while the gray-level distribution of vessel bifurcations is highly asymmetric. Thus, the gradient distributions of nodules and vessel bifurcations are different. To represent gradient distribution accurately, surface gradient features and shell-based gradient features were both extracted.

Surface gradient features were extracted. Firstly, surface voxels of lung nodule candidates were extracted. 26-connected neighborhood was adopted for this research. Then, three 3 × 3 × 3 isotropic convolution kernels were constructed, and the 3D isotropic kernel coefficients were set. The surface voxels were convolved with three 3 × 3 × 3 isotropic gradient kernels, one for each of the *x*, *y*, *z* directions, and components of gradient vectors for each direction, designated *G*_*x*_, *G*_*y*_, *G*_*z*_, were obtained. Finally, these seven features: maximum (*SurG*_max_), minimum (*SurG*_min_), mean (*SurG*_m*ea*n_), standard deviation (*SurG*_*std*_), skewness (*SurG*_*skewness*_), kurtosis (*SurG*_*kurtosis*_), and small value ratio (*SurG*_*svr*_), that is, the percentage of the gradient vector with small magnitude, of the surface gradient magnitude were extracted. The features mean (*SurG*_m*ea*n_), standard deviation (*SurG*_*std*_), skewness (*SurG*_*skewness*_), and kurtosis (*SurG*_*kurtosis*_) of surface gradient magnitude were shown as Eqs 6, 7, 8 and 9.

$$SurG_{mean}=\left(\frac{1}{n}\sum_{i=1}^{n}SurG_{i}\right)$$ (6)

$$SurG_{std}={\left(\frac{1}{n-1}\sum_{i=1}^{n}{\left(SurG_{i}-SurG_{mean}\right)}^{2}\right)}^{\frac{1}{2}}$$ (7)

$$SurG_{skewness}=\frac{\frac{1}{n}\sum_{i=1}^{n}{\left(SurG_{i}-SurG_{mean}\right)}^{3}}{{\left(\sqrt{\frac{1}{n}\sum_{i=1}^{n}{\left(SurG_{i}-SurG_{mean}\right)}^{2}}\right)}^{3}}$$ (8)

$$SurG_{kurtosis}=\frac{\frac{1}{n}\sum_{i=1}^{n}{\left(SurG_{i}-SurG_{mean}\right)}^{4}}{{\left(\frac{1}{n}\sum_{i=1}^{n}{\left(SurG_{i}-SurG_{mean}\right)}^{2}\right)}^{2}}$$ (9)

In the equations mentioned above, *SurG*_*i*_ denotes the surface gradient magnitude of the i^th^ surface voxel while *SurG*_m*ea*n_ represents the mean surface gradient magnitude for all the surface voxels.

Shell-based gradient features[35] were also extracted. The aim of adopting shell-based gradient features was to supplement the surface gradient features, as surface gradient features are susceptible to the accuracy of lung nodule segmentation.

Five shell-based gradient field strength features were extracted, which were average (*ShellGM*_*av*_), standard deviation (*ShellGM*_*std*_), coefficient of variation (*ShellGM*_*cv*_), maximum value (*ShellGM*_max_) and minimum value (*ShellGM*_min_).

Besides the strength features which were extracted, five orientation features of the shell-based gradient field were also used to discriminate nodules from vessel bifurcations. They were the maximum value (*ShellGD*_max_), minimum value (*ShellGD*_min_), median value (*ShellGD*_m*ed*_), squared ratio of the minimum value to the maximum value (ShellGD(minmax)2), and squared ratio of median value to maximum value (ShellGD(medmax)2) of the shell-based gradient field orientation feature.

#### Shape-based features

Lung nodules have different shapes with vessel bifurcations. Six three-dimensional gray-level based features were extracted as follows: 1) compactness, 2) irregularity[7], 3) sphericity, 4) elongation-shape, 5) flatness-shape, and 6) non-compactness.

Features of elongation-shape, flatness-shape, and non-compactness are based on the moment of inertia tensor which describes how the mass of an object is distributed. The moment of inertia tensor of nodule candidate C can be calculated according to Eq 10.

$$I\left(C\right)=\left[\begin{array}{ccc} I_{xx}\left(C\right) & I_{xy}\left(C\right) & I_{xz}\left(C\right)\\ I_{yx}\left(C\right) & I_{yy}\left(C\right) & I_{yz}\left(C\right)\\ I_{zx}\left(C\right) & I_{zy}\left(C\right) & I_{zz}\left(C\right) \end{array}\right]$$ (10)

With

$$I_{xx}\left(C\right)=\sum_{k=1}^{N}m_{k}\left({\left(y_{k}-\bar{y}\right)}^{2}+{\left(z_{k}-\bar{z}\right)}^{2}\right)$$ (11)

$$I_{yy}\left(C\right)=\sum_{k=1}^{N}m_{k}\left({\left(x_{k}-\bar{x}\right)}^{2}+{\left(z_{k}-\bar{z}\right)}^{2}\right)$$ (12)

$$I_{zz}\left(C\right)=\sum_{k=1}^{N}m_{k}\left({\left(x_{k}-\bar{x}\right)}^{2}+{\left(y_{k}-\bar{y}\right)}^{2}\right)$$ (13)

$$I_{xz}\left(C\right)=I_{zx}\left(C\right)=-\sum_{k=1}^{N}m_{k}\left(x_{k}-\bar{x}\right)\left(z_{k}-\bar{z}\right)$$ (14)

$$I_{xy}\left(C\right)=I_{yx}\left(C\right)=-\sum_{k=1}^{N}m_{k}\left(x_{k}-\bar{x}\right)\left(y_{k}-\bar{y}\right)$$ (15)

and

$$I_{yz}\left(C\right)=I_{zy}\left(C\right)=-\sum_{k=1}^{N}m_{k}\left(y_{k}-\bar{y}\right)\left(z_{k}-\bar{z}\right)$$ (16)

Variables in the equations mentioned above are interpreted as below. *I*(*C*) denotes the moment of inertia tensor of nodule candidate C. *I*_*xx*_(*C*), *I*_*yy*_(*C*), and *I*_*zz*_(*C*) indicate rotational inertia around the x, y and z axis, respectively. *I*_*xy*_(*C*) represents the moment of inertia around the x-axis when the object rotates around the y-axis. Similar conclusions can be obtained for *I*_*xz*_(*C*) and *I*_*yz*_(*C*). The index k runs over all the voxels of a lung candidate. (*x*_*k*_, *y*_*k*_, *z*_*k*_) denotes the coordinates of the voxel k. $\left(\bar{x},\bar{y},\bar{z}\right)$ denotes the coordinates of the weighted centroid of nodule candidate C. *m*_*k*_ is the mass of the voxel k, which is equal to the CT density associated with the voxel k (in HU) multiplied by the voxel volume (in mm^3^). As the 3D image of the pulmonary vessel tree group has been made isotropic, with the size of the voxels equal to 1 mm in each dimension in the prior step, *m*_*k*_ is numerically equivalent to the CT density of voxel k. Once each component of the moment-of-inertia tensor is calculated, the moment of inertia tensor for each nodule candidate is obtained.

Then, three features were extracted based on the three eigenvalues of the moment of inertia tensor, including elongation-shape, flatness-shape and non-compactness. The elongation-shape was computed as the ratio of the first two eigenvalues of the moment of inertia tensor, which was sensitive to elongated structures, shown in Eq 17. The flatness-shape was computed as the ratio of the last two eigenvalues of the moment of inertia tensor, which is sensitive to flat or sheet-like objects, shown as Eq 18. The non-compactness was computed as the ratio of the trace of the moment-of-inertia tensor to the volume raised to the power 5/3, which is sensitive to any deviation from spherical shape, shown as Eq 19.

$$elongation-shape=e_{1}/e_{2}$$ (17)

$$flatness-shape=e_{2}/e_{3}$$ (18)

non−compactness=Tr(I(C))V(C)53=e1+e2+e3V(C)53 (19)

In the equations mentioned above, *e*_1_, *e*_2_ and *e*_3_ are the three eigenvalues of the moment of inertia tensor, which satisfy *e*_1_ ≥ *e*_2_ ≥ *e*_3_.

### Random subset feature selection

The classifier’s performance will often degrade with high-dimensional features. Both feature selection and feature extraction can reduce feature dimensionality to improve the performance on a classification task[36].

Feature extraction is a kind of method in which one tries to develop a transformation of the input space into the low-dimensional subspace that preserves most of the relevant information; feature selection is a kind of method in which one selects only those input dimensions that contain the relevant information for solving the particular problem[37, 38].

A drawback of feature extraction is the fact that the linear combination of the original features is usually not interpretable, and the information about how much an original feature contributes is often lost[39, 40]. Thus, feature selection technology was adopted in this research to reduce feature dimensionality by selecting an optimal feature subset. However, it is a challenging task.

After considering classification accuracy and computation time, the Random Subset Feature Selection (RSFS)[41] algorithm was chosen to select the most discriminating features from the feature pool. The RSFS algorithm repetitively chooses a random subset of features from the set of all possible features. Then, it classifies the data with a kNN classifier using these features. During each iteration, relevance of each feature is updated according to the classification performance of the subset that the feature participates in. Each feature is evaluated based on its average usefulness. Finally, the optimal features are chosen from the feature pool by comparing the relevance values of the features to random walk statistics.

### Classification of lung nodules in the vessel tree group using WSVM with imbalance data

The class of false positives is outnumbered by the class of true nodules once the lax threshold was adopted in the prior step. Thus, the performance of the RSFS feature selection may be degraded as the minority class could be easily overlooked. To avoid problems caused by class imbalance, the undersampling approach[42]—which selects the false positives located near the decision boundaries—was adopted. The false positives far away from the decision boundaries are more likely to be classified correctly, whereas false positives near the decision boundaries—i.e. lying close to the true nodules—are more likely to be incorrectly classified. Thus, most discriminating features from the feature pool were selected to discriminate true nodules from false positives.

The method of using an undersampling technique combined with the Weighted Support Vector Machine (WSVM) was adopted to deal with an imbalanced dataset.

Gmean was adopted to avoid poor prediction accuracy for the minority class, shown as Eq 20.

$$Gmean=\sqrt{Sensitivity\times Specifity}$$ (20)

With

$$Sensitivity(TPR)=\frac{TP}{TP+FN}$$ (21)

$$Specifity(TNR)=\frac{TN}{TN+FP}$$ (22)

In the equations mentioned above, *TP*, *TN*, *FP*, and *FN* stand for True Positive, True Negative, False Positive and False Negative, respectively.

Two-layer cross validation was used in this research. Outer K-fold cross validation was adopted to calculate average detection performance of juxta-vascular nodules in the vessel tree group. Inner L-fold cross validation was adopted to estimate parameters of integer imbalanced sample ratio, cost and gamma (SVM parameters), with a grid search method used. When the best average Gmean was obtained based on inner L-fold cross validation, the optimal combination of parameters was found.

## Experiments

In this section, the performance of the proposed scheme was evaluated to validate the effectiveness of the method. The overall performance using the LIDC database was presented and then compared with other existing methods.

### Evaluation methods

The CT scans were obtained from the Lung Image Database Consortium (LIDC), which is a resource available for public use, purposely used for the evaluation of a CAD scheme for lung cancer detection. Setio et al. excluded thick-slice scans (> 2.5 mm) in LIDC and published the list of selected scans on a public website (http://luna.grand-challenge.org/), which contained 888 scans[20]. For this study, 154 thin-slice scans with a total of 204 nodules were used from that public website.

The gold standard reference of this study was defined as the nodules with diameters between 3 and 30 mm annotated by at least two radiologists. Smaller nodules (the nodules in the < 3 mm category) were not included due to their decreased clinical relevance[7, 43]. Meanwhile, non-nodules, nodules with diameters > 3 mm annotated by only 1 or 2 radiologists, and the nodules < 3 mm were classified as irrelevant findings in the evaluation of the Receiver operator characteristic (ROC) curves.

In this experiment, the weighted SVM classifier was constructed with LIBSVM (version 3.21)[44] when lung nodules were discriminated from false positives in the vessel tree group. Then, the nodule detection performance in the vessel tree group was evaluated by ROC curve. After sensitivity, FPs per case, Gmean, area under curve (AUC) values and overall accuracy were obtained, the proposed scheme was eventually implemented on a desktop PC with Inter(R) Core i7-3770 CPU@ 3.40GHz, 16GB RAM, with Matlab R2014a on Windows XP.

### Evaluation of the threshold value during binarization of the nodule-enhanced image

**Fig 5:**
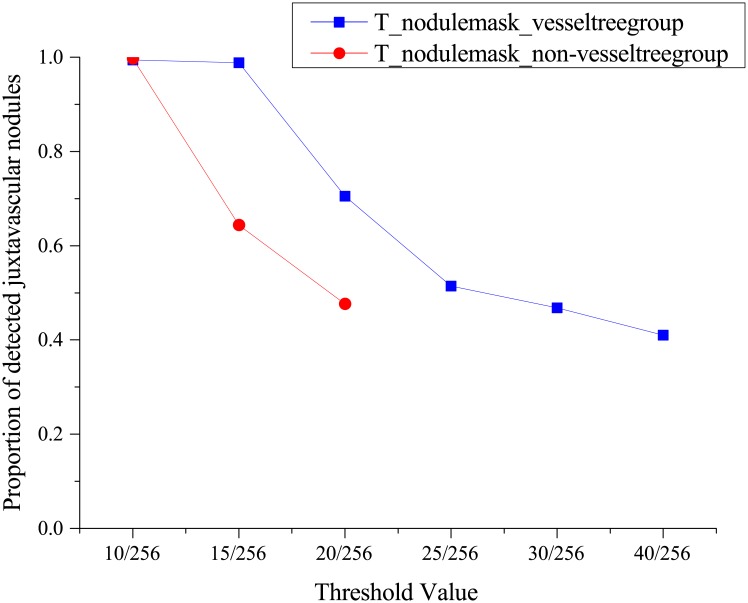
The relationship between the percentage of detected juxta-vascular nodules and the values of *T*_*nodulemask_vesseltreegroup*_ and *T*_*nodulemask_non–vesseltreegroup*_.

The parameter *T*_*nodulemask_vesseltreegroup*_ represented the threshold value during binarization of the nodule-enhanced image of the pulmonary vessels tree group. Similarly, the parameter *T*_*nodulemask_non–vesseltreegroup*_ represents the threshold value to binarize nodule-enhanced images of the non-vessel tree group. If these parameters were set to higher values, more false positives would be removed; meanwhile, subtle nodules would be undetected. The relationship between the proportion of detected juxta-vascular nodules and the values of *T*_*nodulemask_vesseltreegroup*_ and *T*_*nodulemask_non–vesseltreegroup*_ was illustrated in Fig 5. From Fig 5, it can be concluded that most of the juxta-vascular nodules in the vessel tree group and non-vessel tree group could be detected when *T*_*nodulemask_vesseltreegroup*_ and *T*_*nodulemask_non–vesseltreegroup*_ were set to 15/256 and 10/256, respectively. The values were divided by 256 since the pixel values were normalized to the 0–1 range at the window display step.

**Fig 6:**
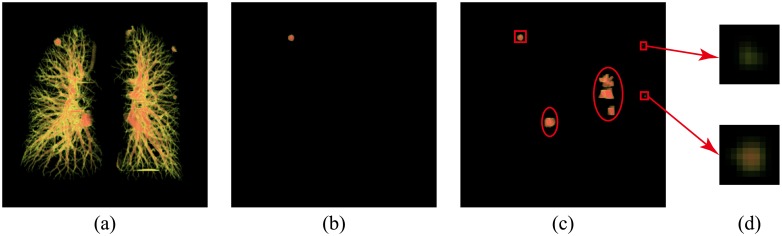
Nodule-enhanced image of the vessel tree group. **a)** 3D image of the vessel tree group, **b)** nodule-enhanced image obtained by using Li’s threshold, **c)** nodule-enhanced image obtained by using the proposed threshold containing nodules in the boxes and vessel bifurcations in the ellipses and **d)** enlarged view of subtle nodules.

The effects of parameter *T*_*nodulemask_vesseltreegroup*_ on juxta-vascular nodule detection in the vessel tree group are illustrated in Fig 6. Fig 6a shows 3D images of the vessel tree group. As shown in Fig 6b, a threshold level of 40—as suggested by Li[7]—would miss some nodules in the LIDC dataset. By setting the parameter *T*_*nodulemask_vesseltreegroup*_ to 15/256, the proposed scheme can detect subtle nodules which were missed by using Li’s threshold, though the vessel bifurcations were also enhanced (See Fig 6c). In Fig 6c, detected nodules and vessel bifurcations detected by mistake were marked in the boxes and in the ellipses, respectively. The enlarged view of subtle nodules is seen in Fig 6d. The vessel bifurcations were removed by using the WSVM classification.

The parameter *T*_*nodulemask_non–vesseltreegroup*_ was set to 10/256. The bifurcations of the tiny vessels—which were also enhanced by dot filter—were not selected because the response of the dot filter was much weaker than that of the vessel tree group.

### Evaluation result

#### Extraction of nodule candidates

**Fig 7:**
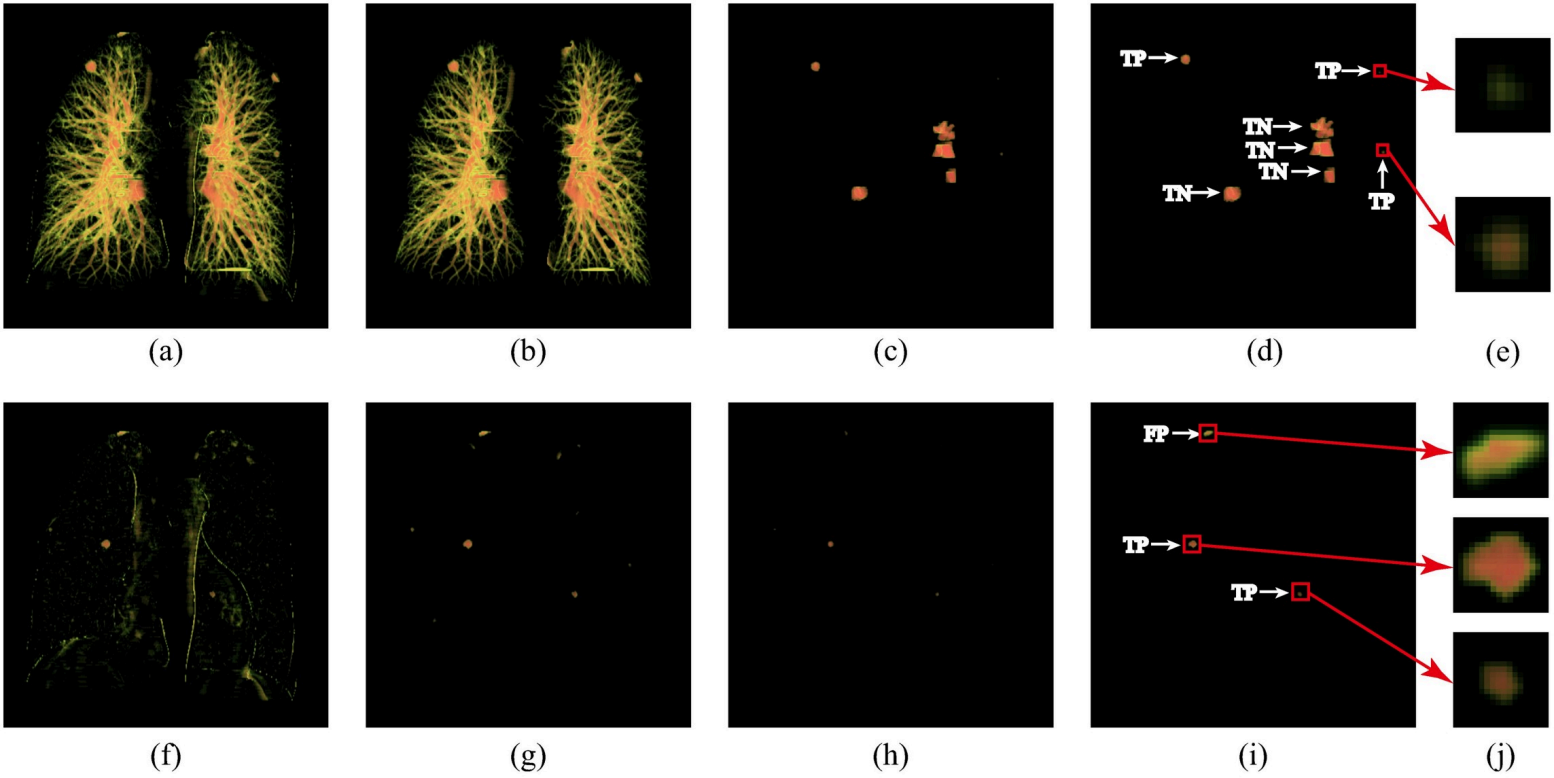
The flow of the nodule candidates’ extraction. **a)** 3D image of all nodule candidates, **b)** 3D image of the vessel tree group, **c)** 3D image after utilizing dot filter in the vessel tree group, **d)** 3D image with the WSVM classification result where lung nodules were classified as true positives and vessel bifurcations were classified as true negatives, **e)** enlarged view of subtle nodules in image d, **f)** 3D image of the non-vessel tree group, **g)** 3D image after removing obvious false positives and noise voxels, **h)** 3D image after utilizing a dot filter in the non-vessel tree group, **i)** 3D image after adopting a constrained region growing method that contained 2 nodules (true positive, TP) with 1 other kind of lesion (false positive, FP) and **j)** enlarged view of nodule candidates from image i.

Fig 6 indicates the flow of nodule candidates’ extraction. Otsu’s method was applied to generate a 3D data set with a high CT value from lung volume, shown in Fig 7a. 3D labeling technology with 26-neighborhood was used to divide the 3D data set into two groups: a vessel tree group, which was shown in Fig 7b, and a non-vessel tree group, which was illustrated in Fig 7f. For the vessel tree group, a dot filter was utilized to extract juxta-vascular nodule candidates, but it also extracted vessel bifurcations, as seen in Fig 7c. Then, the nodule candidates in the vessel tree group were further classified by using WSVM, with the most suitable features selected by RSFS feature selection to eliminate the false positives, as shown in Fig 7d. In Fig 7d, TP and TN represent true nodules and true bifurcations detected by the WSVM classifier. For the non-vessel tree group, a rule-based classifier was implemented to remove obvious false positives and noise voxels, which is shown in Fig 7g. Then, a dot filter was utilized to extract nodule candidates, shown in Fig 7h. Next, objects with volumes smaller than 4.19 mm^3^ were removed. Finally, a constrained region growing method was adopted, as shown in Fig 7i. Fig 7e and 7j represent the enlarged view of subtle nodules and bifurcations, respectively.

#### Feature selection when classifying nodule candidate in the vessel tree group

The most discriminating features selected by the RSFS feature selection algorithm were: *gray_level_max*, *gray_level_min*, *gray_level_mean*, *gray_level_std*, *SurG*_*max*_, *SurG*_*std*_, *SurG*_*skewness*_, *SurG*_*svr*_, *ShellGM*_*av*_, *ShellGM*_*cv*_, *ShellGM*_*min*_, ShellGD(minmax)2, *sphericity*, *irregularity*, *compactness3D*, and *flatness—shape*.

In order to determine the optimal feature subset before selecting the best cross-validation fold combination for the WSVM classifier, an ordinary SVM classifier with balanced training data was constructed.

**Fig 8:**
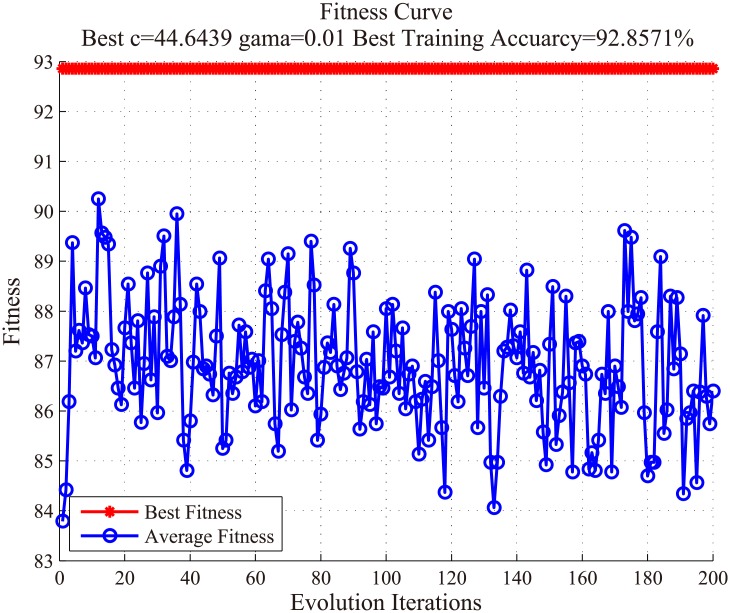
Fitness curve of the PSO algorithm using SVM parameter optimization.

First, 30 positive samples and 30 negative samples were randomly selected to form a training set. Then, the Particle Swarm Optimization (PSO) algorithm was utilized to find the optimal combination of “c” and “gama” parameters with the given features. The fitness curve was drawn when training the selected feature subset mentioned above, as shown in Fig 8. The fitness values in the PSO algorithm—which were evaluated by the fitness function—were continuously optimized through iterations. In Fig 8, the values of both the best fitness and average fitness in the PSO algorithm were satisfactory. When the best values of the c and gama parameters were found, the SVM classifier with optimal parameters was obtained. Using this feature subset selected by the RSFS feature, the training and testing accuracy could reach 92.86% and 92.23%, respectively. This conclusion showed that the selected features mentioned above were highly suitable for lung nodule detection.

#### Performance evaluation of the WSVM classifier with different fold combinations for cross validation

In this research, Radial Basis Function (RBF) was selected as the kernel function of the WSVM classifier. The grid search method was adopted to select the optimal value of the WSVM parameters. The integer sample ratio ranged from 1 to the imbalance ratio, and the imbalance ratio was calculated as the ratio of the negative samples to the positive samples in the training dataset. Additional, the cost parameter of WSVM ranged from 2^−1^, 2^0^, … 2^13^, while the range of gamma was 2^−7^, 2^−5^,…2^−1^,2^0^. The negative samples were selected to compose the modified training data set with positive samples by using the undersampling approach[42], and the number of selected negative samples depended on the integer sample ratio.

As mentioned before, the proposed scheme used a two-layer cross validation method. Different fold combinations were studied to get the best result. Five performance measures were shown in Table 1.

**Table 1:** Performance comparison of different fold combinations based on the five measures.

Different fold combinations	Gmean	AUC	Accuracy	Sensitivity	Specificity
K = 12, L = 7	0.8959	0.9514	0.9049	0.8889	0.9066
K = 12, L = 5	0.8933	0.9508	0.9056	0.8819	0.9080
K = 12, L = 3	0.8916	**0.9545**	0.9089	0.8750	0.9124
K = 10, L = 7	0.8908	0.9465	0.9065	0.8733	0.9101
K = 10, L = 5	0.8973	0.9479	0.9071	0.8867	0.9094
K = 10, L = 3	**0.9020**	0.9477	0.9097	**0.8933**	0.9115
K = 8, L = 7	0.8938	0.9434	**0.9100**	0.8750	0.9138
K = 8, L = 5	0.8914	0.9456	0.9054	0.8750	0.9088
K = 8, L = 3	0.8905	0.9419	0.9100	0.8684	**0.9145**

Best measure results for each fold combination are shown in bold.

As overall accuracy is not a preferred performance measure for imbalanced datasets, Gmean was adopted as a primary evaluation measure in this research. Thus, it is concluded from Table 1 that the best performance of the WSVM classifier is obtained when K = 10, and L = 3.

#### Performance of nodule classification

**Fig 9:**
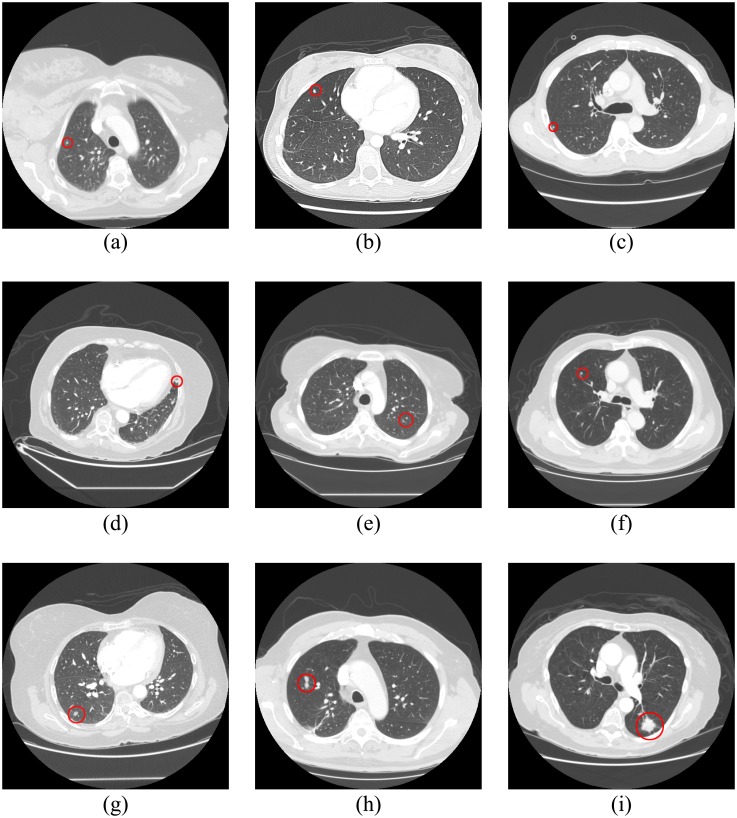
Examples of detected nodules under the proposed scheme, marked with red circles.

The nodule candidates in the non-vessel tree group which survived from the screening technology mentioned above were classified as nodules; meanwhile, the nodule candidates in the vessel tree group classified as true positives by the WSVM classifier were identified as nodules. Fig 9 shows examples of the nodules that were detected by using the proposed scheme. The detailed performance of the nodule classification system was analyzed according to different categories.

For the non-vessel tree group, 115 nodule candidates were detected in the 154 LIDC CT scans, including 77 true positives (63 nodules and 14 micronodules) and 38 FPs. The sensitivity for nodule detection in this group was 92.65% (63/68) and the number of FPs/scan was 0.2468.

**Fig 10:**
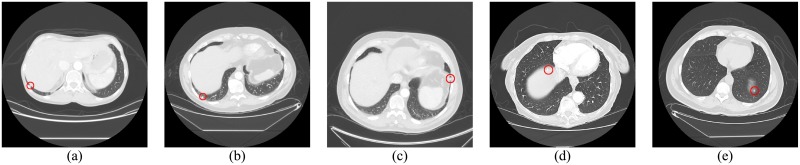
Five undetected nodules in the non-vessel tree group, marked with red circles.

Five lung nodules were missed in the non-vessel tree group. Three nodules attached to pleura were excluded at the lung segmentation step. This was due to special cases where the lung parenchyma was blocked and divided into multiple parts by lung nodules, some of which were possibly mistaken for lung borders. This eventually led to these lung nodules’ being hardly detected and possibly overlooked, all of which is shown in Fig 10a, 10b and 10c. Two other nodules located adjacent to fissures were weakly visible and missed due to low contrast resolution, which is shown in Fig 10d and 10e.

**Fig 11:**
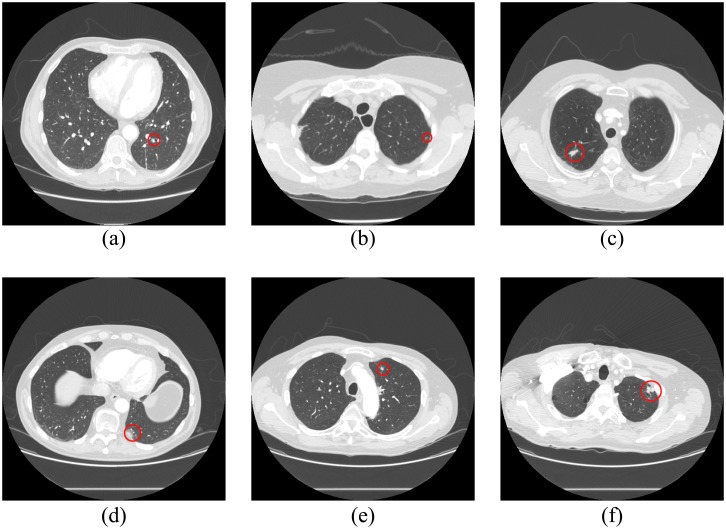
Six undetected nodules in the non-vessel tree group prescreening stage, marked with red circles.

For the vessel tree group, nodule detection was composed of two steps. At the prescreening stage, 1562 nodule candidates were detected in 154 LIDC CT scans, including 153 true positives (130 nodules and 23 micronodules) and 1409 FPs. The sensitivity for detecting nodules in the vessel tree group at the prescreening stage was 95.59% (130/136), and the number of FPs/scan was 9.15. The shape of six missed lung nodules in the vessel tree group was irregular, either because these lung nodules were attached to both the vessel tree and pleural nodules or because they had nodule tails, seen in Fig 11. Thus, these nodules were excluded by dot filter because of their irregular shape. Most of the detected FPs will be removed at the subsequent classification stage.

**Fig 12:**
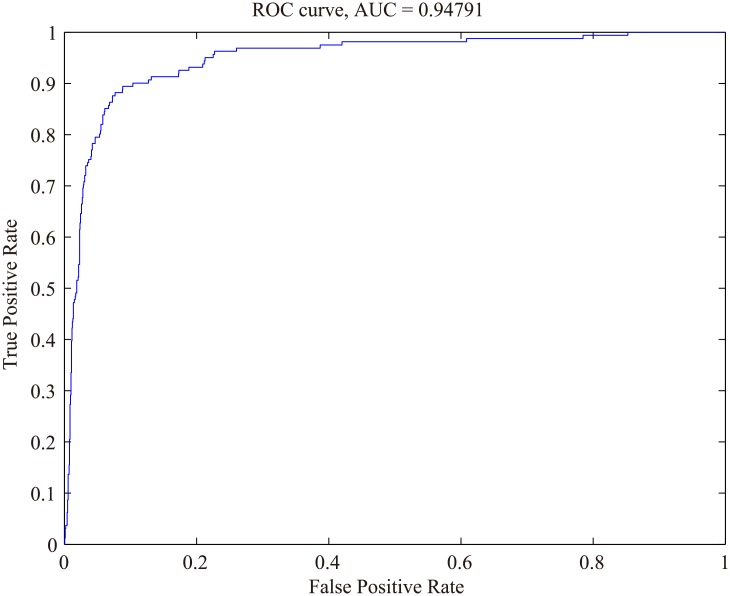
ROC curve of the proposed method dealing with nodule candidates in the vessel tree group.

At the classification stage, the undersampling technique combined with the WSVM classifier was adopted to further remove false positives. The ability to detect nodules in the vessel tree group at the classification stage was represented by the ROC curve, seen in Fig 12. The AUC of the ROC curve was 0.9477, Gmean was 0.9020, and the accuracy was 90.97%, with a false positive rate of 8.85% and 89.33% sensitivity.

The sensitivity for nodule detection in the vessel tree group is the sensitivity of nodule prescreening multiplied by the sensitivity of the WSVM classifier. Similarly, the false positive number for nodule detection in the vessel tree group was the false positive number/scan of the nodule during prescreening multiplied by the false positive rate of the WSVM classifier. Thus, the sensitivity for nodule detection in the vessel tree group is 85.39% and the number of FPs/scan is 0.8098.

In summary, the sensitivity of the proposed CAD scheme is 87.81%($\frac{63+136\times 85.39\%}{204}$), and the number of FPs/scan of the proposed CAD scheme is 1.057.

#### Comparison with the state-of-the-art methods

Two typical types of CAD schemes—conventional method-based and deep learning method-based schemes—were often used for lung nodule detection. Deep learning-based schemes could generally yield a higher performance than conventional method-based schemes. However, these deep learning method-based schemes require a large number of training datasets and a specialized graphics processing unit (GPU) to implement[23]. Thus, a conventional method-based scheme was implemented in this research for lung nodule detection. Twelve recently reported CAD schemes using the LIDC database were chosen for comparison.

Although those limitations (different CT protocols for image datasets, different evaluation methods, and different lung segmentation methods) have an impact on performance evaluation, a relative comparison is still helpful in order to validate the performance of the proposed scheme. The summary of the comparison is presented in Table 2.

**Table 2:** Performance comparison of the proposed scheme with other existing methods.

CAD Scheme	Type of Method	LIDC scans	Nodule Number	Sensitivity	FPs/scan
Xie et al[45]	Deep Learning	888	1186	86.4% 85.2%	4 1
Gu et al[46]	Deep Learning	888	1186	92.9% 87.9%	4 1
Dou et al [47]	Deep Learning	888	1186	93.3% 86.5%	4 1
DeepLung[48]	Deep Learning	888	1186	91.7% 86.5%	4 1
Lu et al [49]	Conventional	98	223	85.2%	3.1
Wang et al [50]	Conventional	103	127	88%	4
Teramoto et al [51]	Conventional	84	103	80%	4.2
Riccardi et al [52]	Conventional	154	117	71%	6.5
Tan et al [19]	Conventional	125	80	87.5%	4
Gong et al[23]	Conventional	888	1186	79.3%	4
MOT_M5Lv1[24]	Conventional	888	1186	81.6%	4
Visia CT Lung CAD[23]	Conventional	888	1186	78.8%	4
Proposed method	Conventional	154	204	87.81%	1.057

As shown in Table 2, the sensitivity of our method is higher than 85%; meanwhile, FPs/scan is lower than most of the existing methods recently reported. When compared with deep learning method-based schemes, the performance of our method is similar to—or slightly higher than—that of Xie’s scheme, Gu’s scheme, Dou’s scheme and DeepLung at 1 FPs/scan. However, these deep learning method-based schemes can yield higher performance than our scheme at 4 FPs/scan. Thus, these deep learning method-based schemes can obtain higher performance. When compared with conventional method-based schemes, our method is similar to Lu’s scheme, Wang’s scheme, and Tan’s scheme in terms of sensitivity, but our method maintained a lower rate of false positives. The proposed scheme gets higher performance than that of Gong’s scheme, MOT_M5Lv1 and Visia CT Lung CAD, but these CAD systems were validated with larger datasets than that of our scheme. It prompts us to test the performance of the proposed scheme with much bigger data sets in the next step. In conclusion, as indicated by Table 2, the performance of our method may be similar to deep learning method-based schemes with low false positive rate and may be better than the existing conventional methods recently reported.

## Discussion

The proposed method has three advantages. The first advantage is that decimals were used to represent gray intensity more accurately. Secondly, different thresholds were used to extract juxta-vascular nodules attached to vessel trees or tiny vessels when nodule-enhanced images were binarized. The final advantage is that the false positive rate of the proposed scheme is much lower than that of the existing methods recently reported, while the sensitivity of the proposed scheme maintained a good performance (87.81%).

At the same time, the proposed method had one limitation. Ground glass opacity nodules were not considered, which would require a modification of the current detection method.

## Conclusion

In this paper, a novel method is proposed to detect lung nodules in chest CT images. The DICOM windows display technology was improved. Then, the lung volume was extracted from the chest CT scan. The nodule candidates were divided into two groups and were detected with different methods. For the non-vessel tree group, the sensitivity of nodule detection was 92.65% with 0.2468 FPs/scan. For the vessel tree group, the sensitivity of nodule detection was 84.76% with 0.8289 FPs/scan. Thus, the proposed CAD scheme detected only 1.076 FPs/scan with 87.46% sensitivity in the LIDC dataset. It could be concluded that the performance of our method may be better than that of the existing methods recently reported. Our future research work will focus on detecting GGO nodules.

## Compliance with ethical standards

This work is done using a public lung CT image database where personal information of patients has been removed. Thus, informed consent is not required. This article does not contain any studies with human participants or animals performed by any of the authors.
